# Direct Reprogramming of Somatic Cells to Neurons: Pros and Cons of Chemical Approach

**DOI:** 10.1007/s11064-021-03282-5

**Published:** 2021-03-05

**Authors:** Cristiana Mollinari, Daniela Merlo

**Affiliations:** 1 Institute of Translational Pharmacology, National Research Council, Via Fosso del Cavaliere 100, 00133 Rome, Italy; 2 Department of Neuroscience, Istituto Superiore di Sanita’, Viale Regina Elena 299, 00161 Rome, Italy

## Abstract

Translating successful preclinical research in neurodegenerative diseases into clinical practice has been difficult. The preclinical disease models used for testing new drugs not always appear predictive of the effects of the agents in the human disease state. Human induced pluripotent stem cells, obtained by reprogramming of adult somatic cells, represent a powerful system to study the molecular mechanisms of the disease onset and pathogenesis. However, these cells require a long time to differentiate into functional neural cells and the resetting of epigenetic information during reprogramming, might miss the information imparted by age. On the contrary, the direct conversion of somatic cells to neuronal cells is much faster and more efficient, it is safer for cell therapy and allows to preserve the signatures of donors’ age. Direct reprogramming can be induced by lineage-specific transcription factors or chemical cocktails and represents a powerful tool for modeling neurological diseases and for regenerative medicine. In this Commentary we present and discuss strength and weakness of several strategies for the direct cellular reprogramming from somatic cells to generate human brain cells which maintain age‐related features. In particular, we describe and discuss chemical strategy for cellular reprogramming as it represents a valuable tool for many applications such as aged brain modeling, drug screening and personalized medicine.

## Commentary

Adult cells are believed to maintain their differentiated status under stable homeostatic conditions, while cellular identity can become plastic when homeostasis is perturbed such as during an injury and inflammation [1]. Indeed, it is now evident that cell identity is more flexible and plastic than previously thought. In particular, recent studies have shown that it is possible to influence cell fate through artificial manipulation such as exogenous expression of a set of transcription factors (TFs) that results in the reprogramming of adult skin fibroblasts to a pluripotent state [2]. In addition, recent reports have demonstrated that one type of differentiated somatic cell can be directly reprogrammed to another type of cell, without rejuvenation to a pluripotent state, in a process called transdifferentiation [3, 4]. Transdifferentiation is an epigenetic acquisition by a cell of a given type of the properties and features of another cell type, loosing its own phenotype [5].

Adult brain has very limited regeneration capability, thus, the possibility of a direct neuronal reprogramming from non-neuronal cells, bypassing a pluripotent state, would induce the formation of precious neuronal cells. This direct cellular generation thus represents a potential remedy for neuronal loss caused by brain injuries or neurodegeneration. In addition, the direct conversion of patient-specific cells could be used to implement disease-relevant in vitro platforms to generate models for neurodegenerative diseases, identify targets, and screen potential therapeutic drugs*.* Indeed, 100s of millions of people worldwide are affected by neurological disorders, making them one of the greatest threats to public health.

**Fig. 1:**
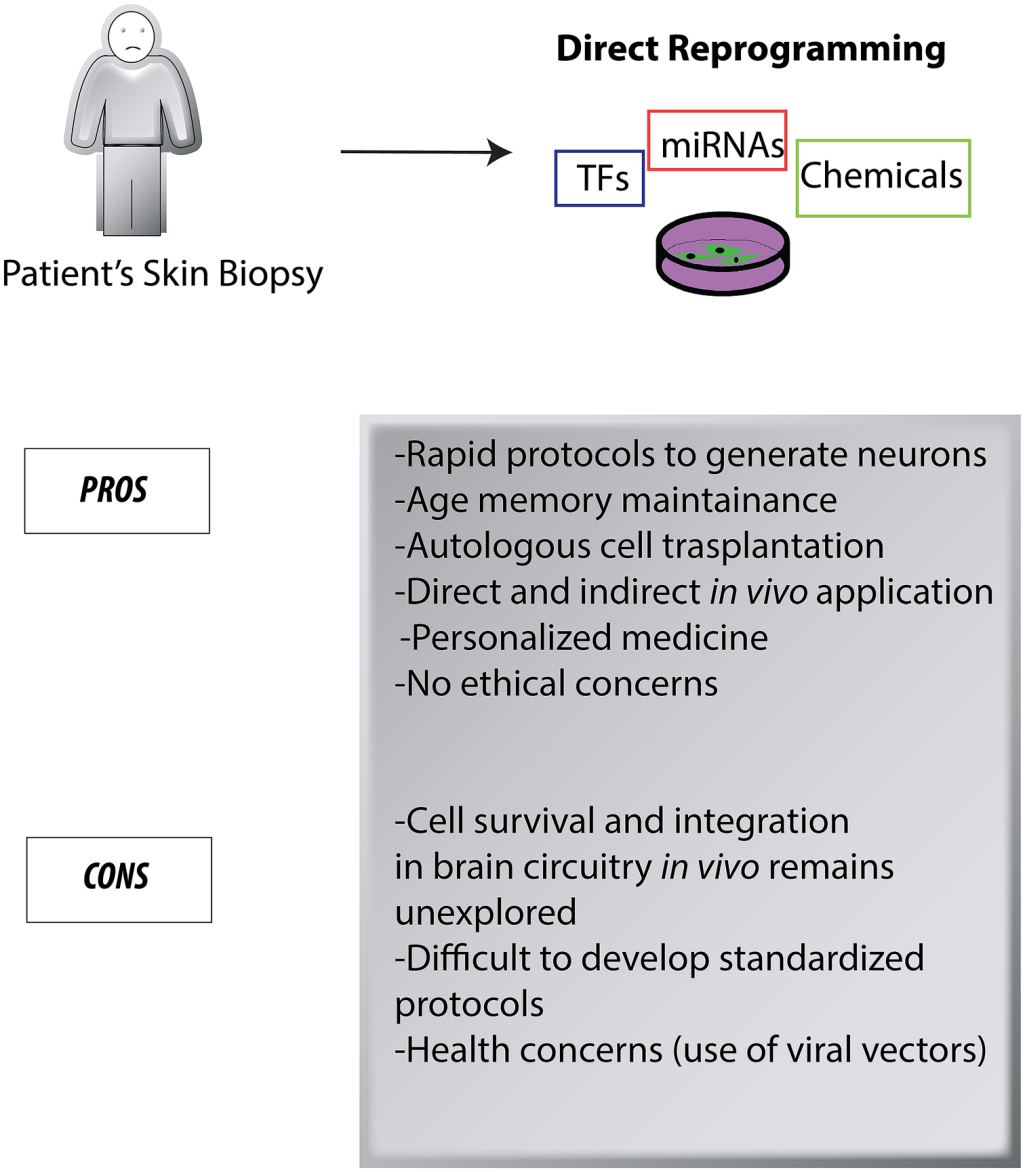
Advantages and disadvantages of direct reprogramming using different exogenous factors

This Commentary discusses current knowledge on direct reprogramming towards neuronal cell identity, and more specifically, recent advances in transdifferentiation mediated by the exclusive use of chemical cocktails, remarking advantages and limits. To our opinion, direct reprogramming approaches represent an innovative strategy to overcome major barrier of the inaccessibility of human brain to obtain human neurons for studies of pathological mechanisms of diseases (Fig. 1). Moreover, directly converted induced neurons (iNs) from human donor-derived fibroblasts possess important features of cellular aging, including global transcriptomic changes, nuclear pore defects, and DNA methylation, rendering them a valuable tool for the study of age-related neurological diseases [3, 6–8]. The importance of age preservation for disease modeling was recently illustrated also in Hungtington’s disease where aggregation of the disease-causing mutant Huntingtin protein can be recapitulated in directly converted striatal neurons but not in neurons derived-iPSC, probably linked to the erasure of age signatures [9].

Among the various strategies to obtain direct reprogramming, ectopic expression of TFs in non-neuronal cells has generated neurons and neural progenitors both in vitro and in vivo [10–26]*.* Direct conversion by TFs stands on their ability to bind to inaccessible neuronal genes in differentiated non‐neuronal cell types which are generally called as pioneer TFs (Fig. 1).

The first direct conversion strategy was achieved by the overexpression of the three TFs, namely Ascl1, Brn2, and Myt1l (BAM factors), in mouse fibroblasts [27], and was then extended to BAM with NeuroD1 to convert human fibroblasts to iNs with a similar efficacy [28]. Recently, it has been suggested that a huge variety of TF combinations can be applied to generate subtype‐specific iNs from fibroblasts (Table 1) and TF screening studies for iN conversion have led to the identification of additional pro‐neuronal factors, such as Brn3a/b/c, Brn4s, and Ezh2 [29, 30]. Particularly interesting appear the recent advances in direct neuronal reprogramming in various defects linked to genetic alterations and ageing such as diabetic retinopathies, glaucoma, and macular degeneration that cause the death of retinal neurons and profound vision loss [31]. Indeed, Lu et al. present evidence that the ectopic expression of OCT4, SOX2 and KLF4 (OSK) TFs safely restores in vivo youthful DNA methylation patterns and transcriptomes of aged retinal ganglion cells and propose that epigenetic reprogramming, either by gene therapy or other means, may promote tissue repair and thus, may be a promising strategy for reverting age-related decline and aged-induced pathologies in humans [32].

**Table 1:** A summary of recent in vitro strategies discussed in this Commentary for direct reprogramming, focusing on chemicals, using human cells

Cell source	Direct conversion strategy	Type of neurons	Methods PROS/CONS	Citations
Fibroblasts	BAM + LMX1a, FOXA2	GLUT + DOPA	Viral vector, health concern	[10]
	BAM + NEUROD1	GLUT	Viral vector, health concern	[28]
	ASCL1, NGN2	GLUT, GABA, DOPA	Viral vector and chemicals High efficiency Health concern	[3, 8, 11, 12, 53, 57]
	Chemical cocktail Valporic acid, Forskolin, Repsox, CHIR99021, SP600125, GO6983, Y-27632	GLUT	Only chemical compounds Low efficiency, no health concern	[49]
	miRNA 9/9*, miRNA 124 ISL1 and LHX3	Motor neurons	Viral vector, health concern	[35]
	miRNA 9/9*, miRNA 124 + TFs (BCL11B, DLX1, DLX2, and MYT1L)	Striatal medium spiny neurons	Viral vector, health concern	[36]
Retinal Müller cells	OCT4, SOX2, KLF4	Retinal neurons with regeneration abilitites and recovered youthful epigenetic information	Viral vector, health concern	[32]
Blood cells	BAM + NGN2	GLUT, GABA	Viral vector, health concern	[13]
Nasal olfactory cells	Chemical and growth factor cocktail BDNF, GDNF, ascorbic acid, cyclic AMP, CHIR99021, NT3, LDN-193189 Noggin, and SB-431542	DOPA	Only chemical compounds Not working on fibroblasts No health concern, Possible application in cell transplants	[60]
Glia cells	Chemical cocktail LDN193189, SB431542, TTNPB, Tzv, CHIR99021, DAPT, VPA, SAG, and Purmo	Mainly GLUT, few GABA	Only chemical compounds No health concern, Works only with glia from brain not spinal cord Fetal human cells, ethical concern	[50]
	Chemical cocktail VPA, CHIR99021, Repsox, Forskolin, i-Bet151, and ISX-9	GLUT	Only chemical compounds No health concern, Adult glia cells, no ethical concern	[51]
	Chemical cocktail SB431542, LDN193189, CHIR99021, and DAPT	GLUT	Only chemical compounds No health concern Fetal human cells, ethical concern	[73]

More interestingly, TFs and endogenous genes vital to the transdifferentiation process can be specifically targeted and silenced or upregulated, using methods that focus on the direct manipulation of DNA or the epigenetic environment, such as CRISPR/Cas9 [33, 34]. Moreover, the ability to drive direct reprogramming is not limited to TFs, as non-coding RNAs can promote it as well [35, 36]. In addition, the culture conditions, including increased time in culture and developing coculture with astrocytes, may have an impact in terms of both phenotypic fate and efficiency of reprogramming.

The use of viral vectors to introduce exogenous transgenes into cells is currently the most prominent method to induce transdifferentiation. Generally, lentiviruses and retroviruses are mostly used due to their ability to effectively integrate directly into the genome of the host cell and confer a proper level of TF expression. However, viral delivery of TFs possesses undesirable side effects, including possible mutations leading to oncogenesis, thus posing problems for possible clinical trial application. That is the reason why non-integrating vectors have been developed, although associated with lower efficiencies of transdifferentiation, including: certain serotypes of AAVs reported to successfully cross the blood–brain barrier, Sendai virus, plasmid vectors, minicircles, and mRNA vectors which remain in the cytoplasm where they are translated into proteins [37–39]. Alternative non-viral methods, such as transient transfection and electroporation (for retina [40, 41], for brain [42–44]) can also be applied. However, due to their low efficiency, transgene silencing, inflammation and poor nuclear uptake, are less commonly used in transdifferentiation studies [45]. Lately, the use of neural exosomes [46] and the protein transduction domains (PTDs) fused to TFs allow the direct delivery of exogenous TFs avoiding the problems associated with DNA integration into the host genome [47] opening up new strategies for possible clinical applications.

Besides TFs, small molecules, modulating specific targets and epigenetic mechanisms, have been used to produce neural progenitors [48] and neurons [49–51] in in vitro cultures (Fig. 1). Small molecules can be applied in combination with viral agent-mediated TF delivery to improve the reprogramming efficiency [52–57] although, chemical reprogramming alone can be easily administrated and converted into therapeutic intervention. In the last years, several groups have identified combinations of small molecules capable of transdifferentiating somatic cells such as fibroblasts, astrocytes and even glioblastoma cells into neurons [48–51, 54, 58] (Table 1). Small molecules can convert human astrocytes or fibroblasts into functional neurons (chemical induced Neurons, ciNs), with a yield of up to 85% neurons from fetal and adult astrocytes [50, 51], which is lower from human fibroblasts, with an efficiency of no more than 15% [49]. For sure, fibroblasts are better starting cells for direct neuronal reprogramming because of easier access for acquisition than astrocytes, although their lower reprogramming efficiency to neurons needs to be increased for broader application in neurological diseases. For example, Yang et al. reported that human fibroblasts can be efficiently and directly reprogrammed into glutamatergic neurons by serially exposing cells to a combination of twelve small molecules [59]. These ciNs displayed neuronal transcriptional networks, and also exhibited mature firing patterns and formed functional synapses. Although many reports have demonstrated that small molecules can convert one type of terminally differentiated somatic cell to another fully differentiated cell type, there are still various major aspects ahead that must be overcome. Indeed, protocols using small molecules produce mainly glutamatergic subtypes with rare gabaergic and dopaminergic neurons (Table 1). The inability to produce the neuronal subtypes which are lost in neurodegenerative disorders like Parkinson’s disease, Alzheimer’s disease, Amyotrophic Lateral Sclerosis, Huntingdon’s disease represents a major limitation in current small molecules transdifferentiation field. However, it was showed that a single TF such as ASCL1, using a novel protein intracellular delivery technology, in combination with the small molecules LDN193189, SB431542, DAPT and valproic acid can rapidly reprogram astrocytes into mature GABAergic and glutamatergic interneurons with high efficiency [47]. Moreover, Chabrat et al. developed a novel in vitro model of dopaminergic-like neurons derived from human nasal olfactory stem cells through a six step transdifferentiation protocol based on a specific combination of signaling pathway modulators [60]. Indeed, chemical cocktails offer the possibility of fine-tuning their effects by altering their concentrations and combinations. Thus, it is reasonable to envisage that by performing screening assays with different small molecules combinations, for example exploiting microfluidic and chip technology, along with slight modifications of the chemical recipe, depending on the starting somatic cell, it would be possible to achieve higher efficiency and additional neuronal lineages.

The main disadvantages of transdifferentiation by chemical approach to generate brain cells with specific properties consist in a low efficiency, a mixed population of neurons with different degrees of maturity and a unique subtype of neurons, although capable to maintain the age-related features associated with the human pathology (Fig. 1). In this respect, it is noteworthy that generation of neurons by direct reprogramming with age and pathology memory, would be important for disease modeling and drug screening studies but would represent a limit for autologous cell transplantation due to the preservation of the pathological features.

Forced expression of exogenous TFs for the direct reprogramming is supposed to damage proper epigenetic marks and genome integrity, whereas chemical compound-based conversion should be milder, leading to a better conservation of the ageing conditions. Thus, we believe that the chemical strategy may represent a new valid method for generating cells for both basic research and clinical applications. It is important to consider that the rapid metabolic transition that takes place during the fate switch from somatic cell to neuron puts enormous stress on the cell, leading to the formation of reactive oxygen species (ROS), known to induce toxicity and affect cell fate regulation, representing a major barrier to transdifferentiation [61]. For this reason an intermediate stage of reprogramming would reduce this oxidative stress, promoting a safer transition between cell fates and improving efficiency [21]. In this respect, the generation of neural stem or progenitor cells (NPCs) from other somatic cells, can largely improve the efficiency of the protocol since each neural stem cell can produce several neurons.

Small molecules can also facilitate the approach of Cell Activation and Signaling-Directed (CASD) reprogramming, which leads cells into an epigenetically activated transition state (cell activation) that, in conjunction with lineage-specific signals (signaling-directed), reprograms somatic cells into NPCs [62–66]. In this respect, Zhu et al. demonstrated that a single gene, Oct4, in conjunction with a chemical cocktail containing CHIR99021, A-83-01, NaB, LPA, rolipram, and SP600125 was sufficient to convert human fibroblasts into expandable NPCs [67].

The most exciting perspective of direct reprogramming is the possibility that it might be achievable in patients in vivo [68–70]*.* Performing in vivo transdifferentiation would eliminate the need for cell transplantation and immunosuppression depending on the target application. However, potential adverse effects of direct reprogramming in vivo could include inappropriate differentiation into other cell types or even tumor cells. In addition, induced cells could be dysfunctional and detrimental to the brain structure. On the other hand, implantation of patient-derived midbrain dopaminergic progenitor cells, differentiated in vitro from autologous iPSCs, was succesfull to stabilize/improve symptoms of PD without the need for immunosuppression [71].

In animal models, transdifferentiation in vivo is now currently feasible, revealing the important role of resident glial cells in the generation of specific neurons to restore lost neuronal circuitries. For example, reactive astrocytes and NG2 cells can be directly reprogrammed into functional neurons inside mouse brain with the expression of a single neural TF, NEUROD1 [19]. Other TFs, such as neurogenin 2 (NGN2), ASCL1, and SOX2, have also been reported to reprogram glial cells into neurons both in vitro and in vivo [72].

Unfortunately, so far, in vivo studies to induce chemical transdifferentiation accomplished only with small molecules resulted either in promoting only an increase in adult brain neurogenesis [73] or reprogramming of mouse astrocytes into scattered functional mature neurons with electrophysiological characteristics and integration with resident neurons in the brain [74]. In complex, current in vivo studies although appealing are still superficial and limited to confirm reprogrammed cell capabilities, cell survival and integration and a more extensive testing in animal models is necessary before finding a clinical application.

In conclusion, over the past years, several strategies for direct cellular reprogramming have been developed to generate brain cells with age‐preserved features rendering them a valuable tool for many applications such as aged brain modeling and age‐related diseases.

Although transdifferentiation methods, due to the low efficiency, are quite limited, there is ongoing research that aims at improving this limit specially with the advent of in situ transdifferentiation, and with the emergence of CRISPR/Cas9 system as an alternative to TF overexpression methods. In addition, although some disadvantages need to be overcome, transdifferentiation by chemical reprogramming remains an important tool not only in vitro for disease modeling, new biomarkers discovery and drug screening, but also for future possible application in regenerative medicine.
